# Leaning on Providence

**Published:** 1876-05

**Authors:** 


### LEANING ON PROVIDENCE.

PRICELESS above all of Ophir's gold, and the long-famed mines of Golconda, is an affectionate and trusting abandonment of one's self, to the disposition and leadings of Him whose loving-kindness is over all his works. Happier far than any king, is the humblest mechanic, "whose mind is stayed, whose heart is fixed, trusting in" the Maker of us all; who, through darkness and storm, in sickness, and in bereavement, in pain of body and mental grief, can lovingly look upward and say: "Though he slay me, yet will I trust in him." Such a man never commits suicide, never becomes a lunatic, never fills a drunkard's grave. This single, simple virtue, of "LEANING ON PROVIDENCE," is the true Balm of Gilead, would soothe more hearts, would quiet more restlessness, would avert more of eating anxiety, and drive out more remorses—groundless, or for cause—than any remedy known to men. This, however, is a medicine which money can never buy; but all can get it, for all that is necessary is to "search the Scriptures," and patiently observe men and things as they are passing. It is the condition of the mind which makes or mars multitudes; and he will be the most successful physician, who best understands its treatment. One of the great errors of the age is: we medicate the body too much—the mind too little. Every thing goes now by steam and electricity. All is hurry-skurry, helter-skelter. "*Neck or nothing*," is practically the universal motto; and to this high-pressure condition, the truly wise will, day by day, interpose the antagonizing influences of a thoughtful reading of the two most valuable of all books, the Book of Grace and the Book of Providence—in plainer words, the Bible and current history.

---

RATS are not the only species of tenants that outwit their landlords; they will sometimes shun all baits and traps. As many modes of getting rid of them cause them to die on the premises and taint the atmosphere, or are dangerous to human life, it may be well to remember, that if the centre of a cage is sprinkled with a few drops of the OIL OF RHODIUM (a species of *Convolvulus*, from the Canary Isles, fifty pounds of the root of which yield one pound of the essential oil, according to LINDLEY), multitudes are irresistibly attracted to the spot, to be disposed of at will.

